# ^18^F-FACBC and ^18^F-FDG PET/MRI in the evaluation of 3 patients with primary central nervous system lymphoma: a pilot study

**DOI:** 10.1186/s41824-024-00189-6

**Published:** 2024-01-31

**Authors:** Trine Husby, Knut Johannessen, Erik Magnus Berntsen, Håkon Johansen, Guro Fanneløb Giskeødegård, Anna Karlberg, Unn-Merete Fagerli, Live Eikenes

**Affiliations:** 1 Department of Circulation and Medical Imaging, Faculty of Medicine and Health Sciences, Norwegian University of Science and Technology, Postboks 8905, Trondheim, Norway; 2 Department of Oncology, St. Olavs Hospital, Trondheim University Hospital, Trondheim, Norway; 3 Department of Radiology and Nuclear Medicine, St. Olavs Hospital, Trondheim University Hospital, Trondheim, Norway; 4 Department of Public Health and Nursing, Faculty of Medicine and Health Sciences, Norwegian University of Science and Technology, Trondheim, Norway; 5 Department of Clinical and Molecular Medicine, Faculty of Medicine and Health Sciences, Norwegian University of Science and Technology, Trondheim, Norway

**Keywords:** PCNSL, PET, MRI ^18^F-FACBC, ^18^F-FDG

## Abstract

**Background:**

This PET/MRI study compared contrast-enhanced MRI, ^18^F-FACBC-, and ^18^F-FDG-PET in the detection of primary central nervous system lymphomas (PCNSL) in patients before and after high-dose methotrexate chemotherapy. Three immunocompetent PCNSL patients with diffuse large B-cell lymphoma received dynamic ^18^F-FACBC- and ^18^F-FDG-PET/MRI at baseline and response assessment. Lesion detection was defined by clinical evaluation of contrast enhanced T1 MRI (ce-MRI) and visual PET tracer uptake. SUVs and tumor-to-background ratios (TBRs) (for ^18^F-FACBC and ^18^F-FDG) and time-activity curves (for ^18^F-FACBC) were assessed.

**Results:**

At baseline, seven ce-MRI detected lesions were also detected with ^18^F-FACBC with high SUVs and TBRs (SUV_max_:mean, 4.73, TBR_max_: mean, 9.32, SUV_peak_: mean, 3.21, TBR_peak_:mean: 6.30). High TBR values of ^18^F-FACBC detected lesions were attributed to low SUV_background_. Baseline ^18^F-FDG detected six lesions with high SUVs (SUV_max_: mean, 13.88). In response scans, two lesions were detected with ce-MRI, while only one was detected with ^18^F-FACBC. The lesion not detected with ^18^F-FACBC was a small atypical MRI detected lesion, which may indicate no residual disease, as this patient was still in complete remission 12 months after initial diagnosis. No lesions were detected with ^18^F-FDG in the response scans.

**Conclusions:**

^18^F-FACBC provided high tumor contrast, outperforming ^18^F-FDG in lesion detection at both baseline and in response assessment. ^18^F-FACBC may be a useful supplement to ce-MRI in PCNSL detection and response assessment, but further studies are required to validate these findings.

*Trial registration* ClinicalTrials.gov. Registered 15th of June 2017 (Identifier: NCT03188354, https://clinicaltrials.gov/study/NCT03188354).

## Background

Primary central nervous system lymphoma (PCNSL) is a rare extra-nodal non-Hodgkin malignancy making up 4–6% of extra-nodal lymphomas and 3–5% of primary brain tumors (Villano et al. 2011; Tang et al. 2011). PCNSL incidence is increasing, particularly in immunocompetent elderly patients, despite immunodeficiency being the only known risk factor (Shiels et al. 2016). In recent decades, treatment with high-dose methotrexate (HD-MTX)-based chemotherapy, with or without radiation therapy, has been the backbone PCNSL patient management. For eligible patients, induction therapy with polychemotherapy consisting of methotrexate, cytarabine, thiotepa, and rituximab (MATRix) regimen is administered. Additionally, consolidation with either whole-brain irradiation, or high dose chemotherapy supported by autologous stem cell transplantation (HDC/ASCT) has shown excellent long-term outcomes with a 7-years overall survival of 70% (Ferreri et al. 2022). Prognosis is still very poor for patients that are primary refractory, not eligible for HD-MTX treatment, or relapse following initial remission (Houillier et al. 2020).

Pre-treatment (baseline) imaging aims to define disease extent throughout the central nervous system and to exclude concomitant systemic disease. Currently, only brain contrast enhanced magnetic resonance imaging (ce-MRI) is recommended for PCNSL lesion detection (Barajas et al. 2021). Correct treatment requires clinicians to accurately diagnose and distinguish PCNSLs from other brain lesions such as high-grade gliomas (HGG) and brain metastases (BM), which can appear similar with contrast uptake on conventional MRI, due to a common blood brain barrier (BBB) disruption (Okada et al. 2012). Histopathological diagnosis with cytology/flow cytometry of cerebrospinal fluid, or brain biopsy are therefore a pre-treatment necessity. In addition, intermittent and post-treatment response assessment with ce-MRI is essential for determining treatment strategies and achieving optimal patient outcomes.

MRI is a versatile tool which does not expose patients to radiation and provides excellent soft tissue contrast for the delineation of brain lesions. However, conventional MRI has limitations; contrast agents do not cross the intact BBB, treatment related changes are often not discernible from viable tumor tissue, and MRI can be deficient for measuring nuances in the metabolic environment of tumors. The International Primary CNS Lymphoma Collaborative group (IPCG) recommends a PCNSL 3T MRI protocol consisting of several MR sequences (diffusion weighted imaging, contrast enhanced T1 (ce-MRI) and T2, as well as FLAIR and dynamic susceptibility contrast perfusion), and whole-body positron emission tomography (PET) with ^18^F-2-fluoro-2-deoxy-D-glucose (^18^F-FDG) to rule out systemic disease (Barajas et al. 2021). PET has also shown promise in brain tumor detection and diagnosis. The most established PET tracer ^18^F-FDG can diagnose PCNSLs with high accuracy (Zou et al. 2017). Detection is possible since ^18^F-FDG is a glucose analogue which accumulates in the highly metabolic PCNSLs, and uptake intensity likely correlates with tumor cell density (Okada et al. 2012). Still, healthy brain tissue has a high baseline glucose consumption, leading to unfavorable ^18^F-FDG PET brain lesion contrast (Schaller 2004). Functional imaging with brain ^18^F-FDG PET is therefore not established as standard modality for lesion detection at baseline or to evaluate chemosensitivity and to guide treatment strategies in response assessment in PCNSL in contrast to systemic Hodgkins lymhoma and aggressive non-Hodgkin lymphoma. There is an increased interest in PET tracers that target other aspects of brain tumor cell biology, such as amino acid PET (AA-PET) tracer uptake which coincides with increased expression of AA transporters in malignant tissue.

Static and dynamic AA-PET has shown great promise in detecting brain tumor malignancy and extent, in some cases allowing for earlier detection of tumor progression and treatment response than MRI (Unterrainer et al. 2016; Galldiks et al. 2013). AA-PET tracers 3,4dihydroxy-6-[^18^F]-fluoro-l-phenylalanine (^18^F-FDOPA), [^11^C]-methyl-methionine (^11^C-MET), and [^18^F]-fluoro-ethyltyrosin (^18^F-FET) are recommended by international guidelines to complement MRI in the evaluation of gliomas (Albert et al. 2016). Nevertheless, the response assessment in Neuro-oncology working group (RANO) does not currently recommend AA-PET for the diagnosis and response assessment of PCNSLs, owing to a scarcity of studies and consequently limited evidence (Barajas et al. 2021).

Thus far, the only AA-PET PCNSL studies with multiple subjects utilize ^11^C-MET (Okada et al. 2012; Postnov et al. 2022; Jang et al. 2012; Ahn et al. 2018; Kawase et al. 2010; Nomura et al. 2018; Takahashi et al. 2014; Ogawa et al. 1994; Kawai et al. 2010; Miyakita et al. 2020), and there are few published PCNSL cases using ^18^F-FDOPA and ^18^F-FET PET (Puranik et al. 2019; Salgues et al. 2021). The aforementioned AA-PET tracers rely primarily on the L-amino-acid-transporter 1 (LAT1) for accumulation in cancerous cells (Heiss et al. 1999; Sun et al. 2018; Habermeier et al. 2015; Ono et al. 2013). ^18^F-FACBC is another AA-PET tracer that also utilizes LAT1, though it has a higher affinity for the alanine-serine-cysteine transporter 2 (ASCT2) (Ono et al. 2013; Okudaira et al. 2013; Pickel et al. 2020). This feature results in a characteristic low background ^18^F-FACBC uptake and high lesion contrast, as demonstrated in glioma and BM studies (Tsuyuguchi et al. 2017; Kondo et al. 2016; Wakabayashi et al. 2017; Michaud et al. 2020; Bogsrud et al. 2019; Karlberg et al. 2017, 2019; Johannessen et al. 2021; Parent et al. 2020; Shoup et al. 1999; Øen et al. 2022), nevertheless ^18^F-FACBC is not yet recommended by international guidelines for the assessment of brain tumors.

The aim of this pilot study was to evaluate the added value of ^18^F-FACBC and ^18^F-FDG, as a supplement to standard clinical practice, ce-MRI, in terms of lesion detection at baseline and in response assessment in PCNSL patients. In addition, SUVs were assessed for ^18^F-FACBC and ^18^F-FDG, and time-activity curves were assessed for ^18^F-FACBC.

## Methods

### Patients

From November 2017 to September 2021 three PCNSL patients newly diagnosed at St. Olav’s hospital, Trondheim University Hospital (Trondheim, Norway) were included in the study (Table 1). Inclusion criteria were immunocompetence, histological confirmed diffuse large B-cell lymphoma (DLBCL) based on cytology/flow cytometry of CSF or brain biopsy, age ≥ 18 years, and no previous chemotherapy. Corticosteroid treatment was permitted up to 24 h prior to the PET/MRI examinations.

**Table 1:** Patient overview

Patient number	1	2	3
Age	81	75	64
Sex	Female	Female	Male
Histology	DLBCL	DLBCL	DLBCL
Number of lesions	1	5 (plus 1 pituitary adenoma)	1
Biopsy location	Right basal ganglia	Right nucleus lentifomis	Right basal ganglia
First line treatment	R-MPV × 5 + maintenance Temozolomide	R-MPV × 7 + maintenance Temozolomide	MATRix × 4 + BCNU/Thiotepa ASCT
MRI evaluation after 4 or 5 cycles of treatment	Complete remission	Partial remission	Partial remission
Relapse	yes (13 months after initial diagnosis)	yes (4 months after initial diagnosis)	No (16 months after initial diagnosis)
IELSG score	3	4	4
MSKCC score	2	3	3
ECOG PS	2	2	2

*DLBCL* Diffuse large B-cell lymphoma, *R-MPV* rituximab, methotrexate, procarbazine and vincristine,* MATRix* methotrexate, cytarabine, rituximab, thiotepa*, BCNU ASCT* carmustine-thiotepa-conditioned autologous stem cell transplantation,* IELSG score* International Extranodal Lymphoma Study Group score,* MSKCC score* Memorial Sloan Kettering Cancer Center score,* ECOG PS* Eastern Cooperative Oncology Group performance scale

Study inclusion did not alter routine patient clinical data evaluation and treatment led by experienced oncologists, nuclear medical physicians, and neuroradiologists. All patients gave written informed consent, and the study was approved by the Regional Committee for Medical Research Ethics Central Norway (REK, reference number, 2017/325) and the Norwegian Medicines Agency (EudraCT nr: 2017-000306-38).

### PET/MRI acquisitions

Patients were examined with ^18^F-FACBC- and ^18^F-FDG- PET/MRI (Siemens Biograph mMR, Siemens Healthcare, Erlangen, Germany) on two consecutive days, no more than three days before starting treatment (baseline). Response assessment scans were done 7–14 days after five cycles of treatment with rituximab, HD MTX, procarbazine and vincristine (R-MPV) or 14–21 days after four cycles of MATRix. In response assessment, ^18^F-FDG PET/MRI was acquired within four days of ^18^F-FACBC PET/MRI.

### ^18^F-FACBC PET/MRI

The MR protocol included T2, DWI, 3D FLAIR, 3D T1 before and after intravenous contrast, and ultra-short echo time (UTE) for attenuation correction of PET data. The patients were required to fast for a minimum of 4 h prior to the injection of ^18^F-FACBC (mean 220 MBq, range 191–254). The intravenous tracer was injected at t = 0 of a 45-min dynamic PET acquisition. Static PET reconstructions were generated from the last 15 min of the list-mode data, while the dynamic reconstructions were performed with time frames of 12 × 5 s, 6 × 10 s, 6 × 30 s, 5 × 60 s, and 5 × 300 s. PET image reconstruction was performed on the mMR with iterative reconstruction (3D OSEM algorithm, 3 iterations, 21 subsets, 344 × 344 matrix, 4-mm Gaussian filter) with point spread function, decay, and scatter correction. MR-based attenuation correction was performed with a deep learning-based method (DeepUTE) using the ultra-short echo time MR sequence as input for making modified MR-based AC maps (Ladefoged et al. 2020).

### ^18^F-FDG PET/MRI

MRI sequences acquired were UTE for attenuation correction of PET data and 3D FLAIR. Patients fasted for minimum 6 h prior to the injection of ^18^F-FDG (mean 200 MBq, range 178–284) 40-min prior to the start of the PET scan, and a 20-min dynamic/list mode uptake was acquired. Static PET reconstruction was done using the same parameters as for ^18^F-FACBC and was generated from the whole 20 min uptake.

### Clinical image evaluation

Lesion detection was defined separately by clinical evaluations confirming contrast enhanced lesions on MRI and visual assessment of ^18^F-FACBC and ^18^F-FDG PCNSL PET tracer uptake by an experienced neuroradiologist (7 years of experience) and a nuclear medicine physician (8 years of experience), respectively.

### Static FDG and FACBC analysis

PMOD software (version 4.302, PMOD Technologies LLC, Zürich, Switzerland) was used for analysis of all PET data.

Spherical volumes of interest (VOIs) were placed manually to encompass lesions with visible ^18^F-FACBC and ^18^F-FDG tracer uptake to quantify maximum lesion SUV_max_ in the baseline and response scans.

**Fig. 1:**
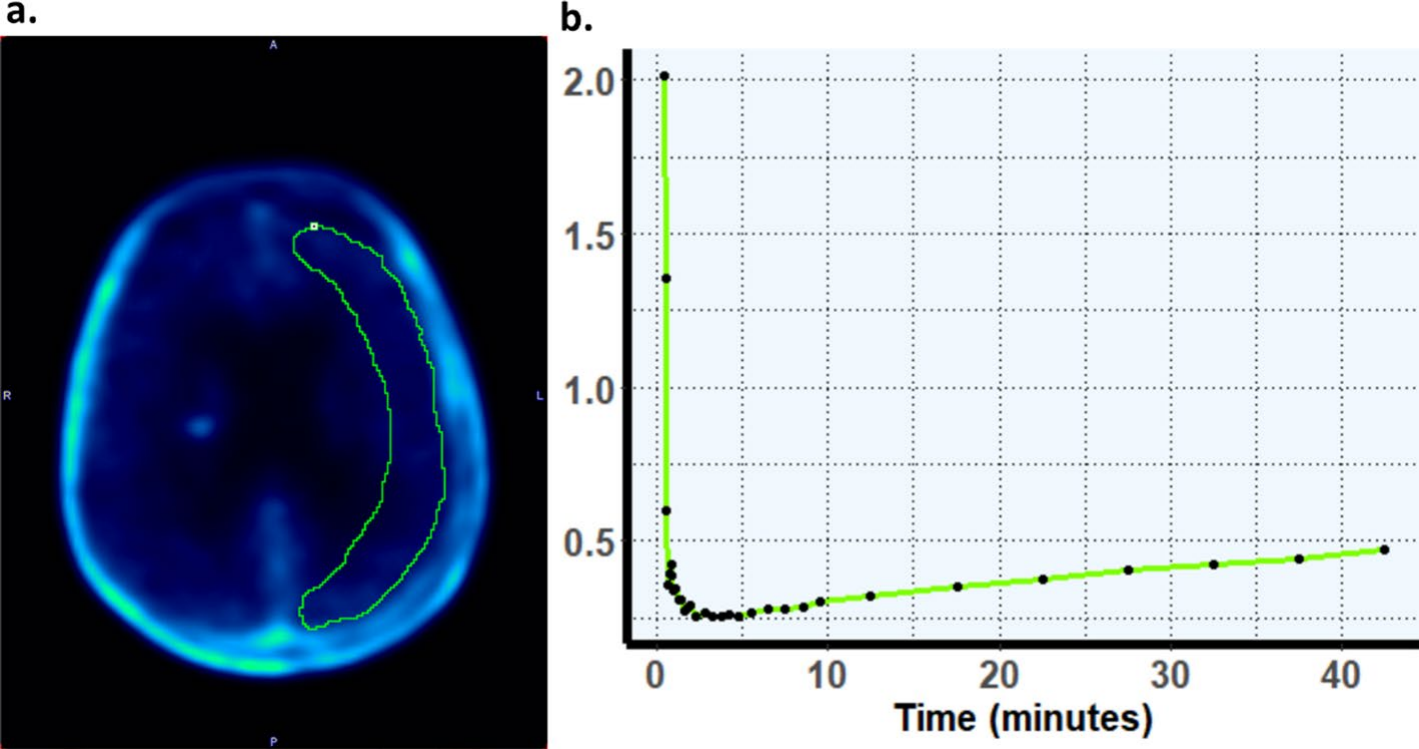
Dynamic ^18^F-FACBC uptake from normal brain tissue. **a** Example of a crescent shaped VOI placed in healthy tissue in the contralateral hemisphere (taken from PET/MR scan from patient 1). **b** Time activity curves (TACs) for SUV_background_ uptake from the baseline scan of patient 1

For ^18^F-FACBC, an additional 1 ml spherical VOI was auto-generated within lesion tracer uptake regions to calculate peak tracer uptake (SUV_peak_), and a crescent shaped VOI was placed on the contralateral side of the brain, to calculate the mean tracer uptake in normal brain tissue (SUV_background_) (Unterrainer et al. 2017) (Fig. 1). For ^18^F-FACBC, tumor-to-background ratios (TBR_max_ and TBR_peak_) were calculated by dividing SUV_max_ (or SUV_peak_), by SUV_background_.

### *Dynamic *^*18*^*F-FACBC analysis*

Spherical VOIs from the static ^18^F-FACBC analysis were used to generate time-activity-curves (TACs) for SUV_background_, SUV_peak_ and TBR_peak_, according to guidelines (Law et al. 2019). TACs were plotted for ^18^F-FACBC detected lesions for the entire 45 min of the scan, starting at 22.5 s after injection to avoid noise.

## Results

### Patient 1

**Fig. 2:**
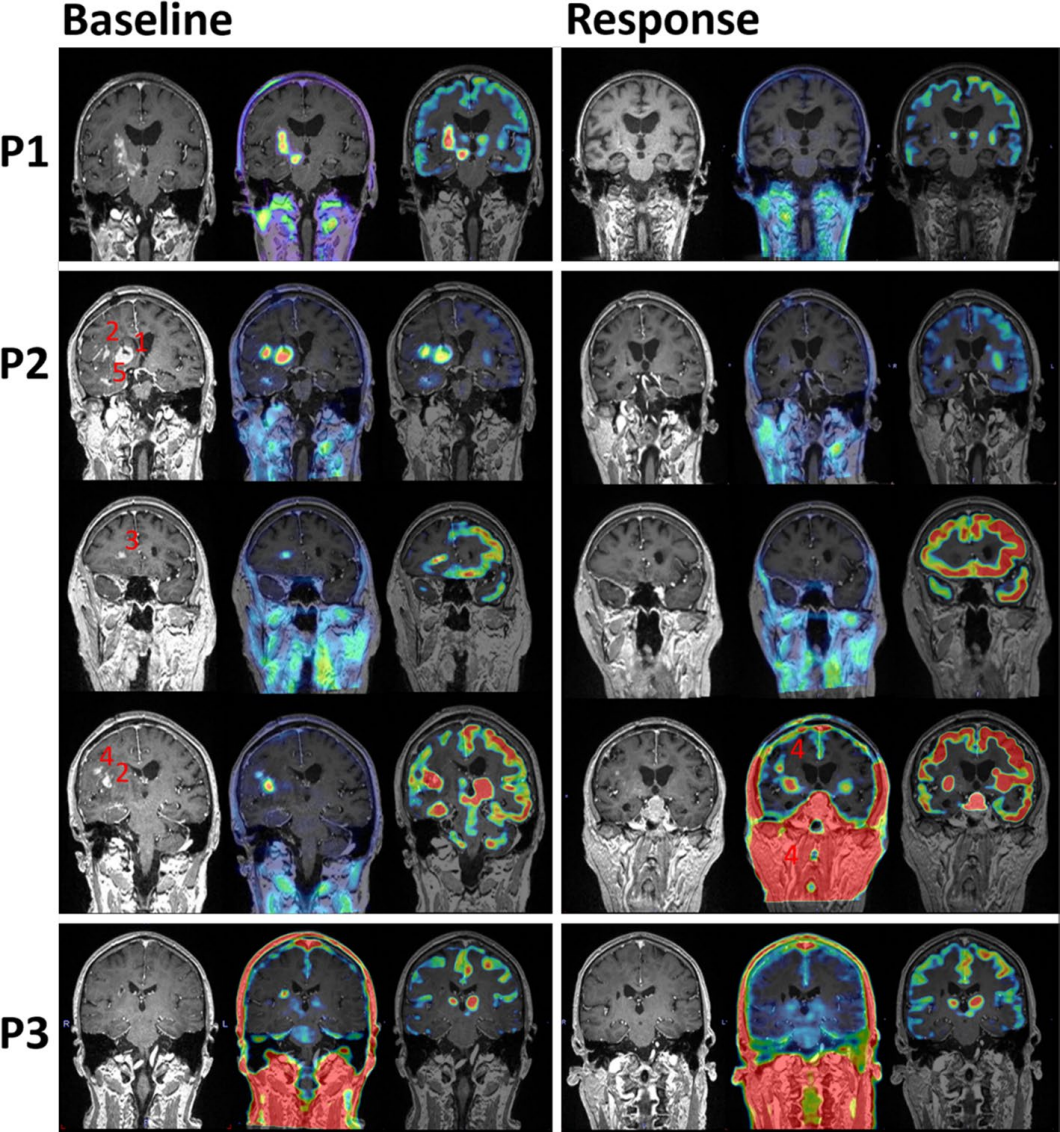
T1 Ce-MRI, fused ^18^F-FACBC/ce-MRI, and fused ^18^F-FDG/ce-MRI are shown from left to right. The left and right panel show baseline and response PET/MRI images, respectively. Patients 1–3 (P1–3) are distributed vertically. Scale for ce-MRI was 0–600. Scale for PET detected lesions was SUV_background_ − SUV_max._ Scale for PET undetected lesions was SUV_background_—SUV_max_ (baseline lesion uptake). For P3 ^18^F-FDG scans, the lesion was undetected in both scans and the scale used was SUV_background (mean)_ − SUV_background (max)_

The 81-year-old female patient presented with headache and left side weakness. Brain MRI showed a solitary lesion in the right basal ganglia and neurosurgical biopsy confirmed DLBCL. At baseline, the lesion (P1(B1)) was detected with ce-MRI, with high tracer uptake on both ^18^F-FDG and ^18^F-FACBC (Table 2, Fig. 2).

**Table 2:** Static PET results from all three patients (P1–3) at baseline (B) and response (R) for PET detected lesions

Patient (lesion)	^18^F-FACBC										^18^F-FDG	
	SUV_background_		SUV_max_		TBR_max_		SUV_peak_		TBR_peak_		SUV_max_	
	B	R	B	R	B	R	B	R	B	R	B	R
P1(1)	0.44	0.39	4.54		10.22		2.81		6.33		8.20	
P2(1)	0.53	0.48	8.37		15.75		6.84		12.87		21.57	
P2(2)			8.90		16.74		5.90		11.10		25.09	
P2(3)			3.57		6.71		1.78		3.36		8.74	
P2(4)			2.78	0.97	5.22	2.02	1.93	0.69	3.64	1.43	8.05	
P2(5)			3.95		7.44		2.50		4.70		11.61	
P3(1)	0.33	0.21	1.03		3.17		0.68		2.08			

The P3 lesion was not detected at baseline with ^18^F-FDG

The patient was treated with five cycles of age-adjusted R-MPV before maintenance with Temozolomide. No lesions were detected in the patient’s ce-MRI, ^18^F-FDG or ^18^F-FACBC in the response assessment scans. Recurrence occurred 13 months after the initial diagnoses.

### Patient 2

The 75-year-old female patient presented with headache, nausea and personality changes. MRI revealed five lesions (P2(B1–5)) in the white matter in the right cerebral hemisphere (Fig. 2), in addition to a pituitary adenoma which was not included in the study. Biopsy from the right lentiform nucleus revealed DLBCL. All five lymphoma lesions were detected with ce-MRI, ^18^F-FDG and ^18^F-FACBC. The small lesion, P2(B4) was difficult to discern with ^18^F-FDG, though it could be detected with knowledge from MRI and ^18^F-FACBC images (Table 2). The patient underwent seven cycles of R-MPV before maintenance with Temozolomide.

In the response scan after five cycles, only lesion P2(R4) was detected with ce-MRI and ^18^F-FACBC, no lesions were detected with ^18^F-FDG. The patient progressed four months after initial diagnoses.

### Patient 3

The 64-year-old male patient presented with dizziness, headache, and cognitive impairment. MRI showed a solitary lesion in the right basal ganglia (Fig. 2). Histology from the neurosurgical biopsy showed DLBCL. At baseline, this small lesion was detected with MRI and ^18^F-FACBC (P3(B1)), but not ^18^F-FDG. However, due to recent biopsy it was unclear whether ce-MRI and ^18^F-FACBC uptake was due to inflammation or malignancy. Due to age and no comorbidity, the patient underwent four cycles of MATRix regime before consolidation with HDC/ASCT.

In the response scan (after four cycles of MATRix), although difficult to distinguish inflammation from malignant tissue, a small lesion was delineated with MRI; the lesion was not detected with ^18^F-FACBC and ^18^F-FDG. The patient was still in complete remission one year after the end of treatment.

### ***Static ***^***18***^***F-FACBC PET/MRI***

All seven ce-MRI detected lesions in the baseline scans were also detected with ^18^F-FACBC, and generally showed high ^18^F-FACBC SUV and TBR (SUV_max_: mean, 4.73; range, 1.03–8.90; TBR_max_: mean, 9.32; range, 3.17–16.74; SUV_peak_: mean, 3.21; range, 0.68–6.84; TBR_peak_:mean, 6.30; range, 2.08–12.87). The high TBR values of ^18^F-FACBC detected lesions were attributed to consistently low SUV_background_ (mean, 0.40; range, 0.21–0.53).

In the response scans, lesions P2(R4) and P3(R1) were detected with ce-MRI, while only one of these lesions (P2(R4)) was detected with ^18^F-FACBC (SUV_max_: 0.97, TBR_max_: 2.02, SUV_peak_: 0.69, TBR_peak_: 1.43).

### ***Static ***^***18***^***F-FDG PET/MRI***

At baseline, the six lesions from P1-2 were detected with ^18^F-FDG with high tracer uptake (SUV_max_: mean, 13.88; range, 8.05–25.09). The solitary ce-MRI and ^18^F-FACBC detected lesion P3(B1) was not detected with ^18^F-FDG at baseline.

No lesions were detected with ^18^F-FDG in the response scans.

### D*ynamic *^*18*^*F-FACBC PET/MRI*

**Fig. 3:**
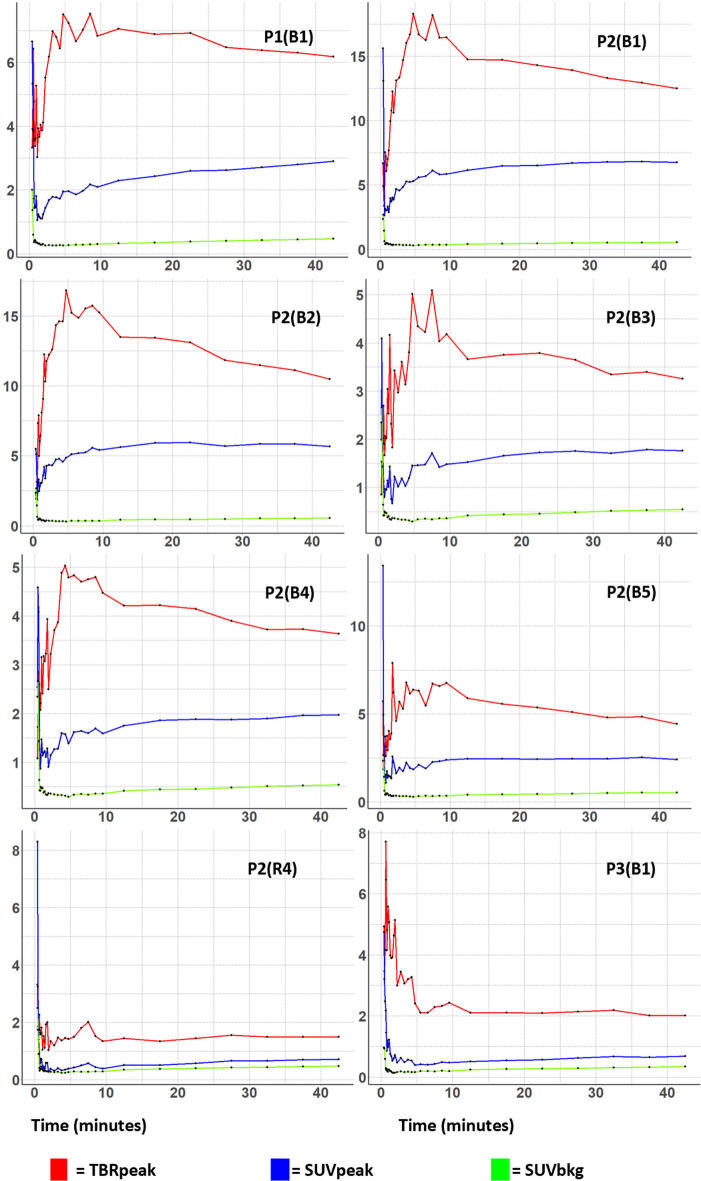
Dynamic time-activity curves for ^18^F-FACBC PET detected PCNSLs. TBR_peak_, SUV_peak_ and SUV_background_ are plotted against time for patients 1–3 (P1–3), for baseline and response scans (B and R). The plot starts at 22.5 s to avoid noise

TACs for SUV_peak_, TBR_peak_, and SUV_background_ of ^18^F-FACBC detected lesions are shown in Fig. 3. All TACs had time-to-peak (TTP) within ten minutes. Following TTP, P1(B1), P2(B1), P2(B3), P2(B2), P2(B3), P2(B4), and P2(B5) all had a decreasing TBR_peak_ curve, whereas P3(B1) and P2(R4) TBR_peak_ TACs plateaued.

## Discussion

This study aimed to evaluate the use of ^18^F-FACBC PET/MRI examination compared to ^18^F-FDG PET/MRI as a supplement to standard clinical practice in patients with PCNSLs, at baseline and for treatment response assessment. This is to our knowledge the first study describing the use of ^18^F-FACBC in this patient group. The rarity, aggressiveness, and accompanying urgency to start treatment of PCNSLs makes it difficult to design studies with an adequate number of subjects, making the literature on this patient group somewhat limited.

In this study, ^18^F-FACBC detection correlated with ce-MRI for all lesions, except one small lesion (P3(R1)) on ce-MRI, which could represent inflammation or disrupted BBB. ^18^F-FACBC outperformed ^18^F-FDG in the detection of PCNSLs, with higher lesion contrast (Fig. 2 and Table 2). The high ^18^F-FACBC lesion contrast was a result of a very low ^18^F-FACBC uptake in normal brain tissue, consistent with findings from previous BM and glioma studies (Tsuyuguchi et al. 2017; Kondo et al. 2016; Wakabayashi et al. 2017; Michaud et al. 2020; Bogsrud et al. 2019; Karlberg et al. 2017, 2019; Johannessen et al. 2021; Parent et al. 2020; Shoup et al. 1999; Øen et al. 2022). This is likely due to slightly different ^18^F-FACBC transport mechanisms across the BBB compared to other AA-PET tracers. ^18^F-FET, ^11^C-MET, and ^18^F-FACBC all utilize LAT1, however, ^18^F-FACBC also utilizes and has a higher affinity for ASCT2 (Ono et al. 2013; Okudaira et al. 2013; Pickel et al. 2020). The distribution of the different transport systems may play a critical role in the differences in AA-PET tracer uptake in healthy tissue. ASCT2 transporters are not expressed on the luminal side of the BBB but are expressed on the abluminal side, possibly resulting in increased luminal ^18^F-FACBC uptake from brain tissue, reducing the tracer concentration in normal brain parenchyma.

The small sample size of this study thwarts the possibility of drawing conclusions with regards to the efficacy of ^18^F-FACBC compared to ^18^F-FDG PET/MRI in baseline detection of PCNSLs, though visual assessment of PET tracer uptake revealed that ^18^F-FACBC produced higher lesion contrast and detected one more lesion (P3(B1)) than ^18^F-FDG. A meta-analysis pooling 129 immunocompetent PCNSL patients from eight retrospective primary research studies reported high diagnostic accuracy using ^18^F-FDG, describing a pooled in-patient sensitivity and specificity of 0.88 and 0.86, respectively (Zou et al. 2017). This high sensitivity and specificity of ^18^F-FDG in PCNSL detection is likely due to PCNSLs having a predilection for localizing in periventricular white matter, and white matter consumes approximately one third of the energy of gray matter (Harris and Attwell 2012). The authors of the meta-analysis conclude that ^18^F-FDG can be a useful diagnostic tool for PCNSL evaluation, and for discerning between PCNSLs, GBMs and BMs through the use of SUV_max_ thresholds (Zou et al. 2017); though localised biopsy is still the gold standard for diagnostic confirmation. Static analysis with AA-PET tracers for the detection of PCNSLs may be superior to ^18^F-FDG, particularly in highly metabolic brain regions, due to a lower SUV_background_ and thus higher lesion contrast. Future studies could assess whether ^18^F-FACBC can be used to distinguish PCNSL malignancy from other tumor types and treatment related changes.

The literature on AA-PET tracer analysis of PCNSLs is very limited, with ^11^C-MET being the only AA-PET tracer reported in studies with multiple subjects. There have been several studies comparing ^11^C-MET to ^18^F-FDG in the evaluation of PCNSLs (Okada et al. 2012; Jang et al. 2012; Kawase et al. 2010; Takahashi et al. 2014; Kawai et al. 2010). Kawase et al. found that although ^18^F-FDG had higher PCNSL lesion uptake than ^11^C-MET, both tracers had similar sensitivity for PCNSL detection because there was no significant difference in TBR (Kawase et al. 2010). Additionally, Jang et al. examined the efficacy of ^11^C-MET PET/CT pre- and post-HD-MTX treatment in four PCNSL patients (Jang et al. 2012). They found that both ^18^F-FDG and ^11^C-MET PET/CT demonstrated clear pre-treatment demarcation of PCNSL lesions, though in one patient ^11^C-MET detected more lesions than ^18^F-FDG. Jang et al. did not detect any lesions in response scans with PET, though MRI showed non-enhancing T2 hyperintensities in one patient, which was truly indicative of residual DLBCL (Jang et al. 2012). Interestingly, a patient (P3(R1)) in the current study also had an MRI equivocal lesion without ^18^F-FACBC uptake in response scans, while the patient was still in complete remission one year after end of treatment. In contrast, the patient that had ^18^F-FACBC uptake in response scans (P2(R4)) progressed four months after initial diagnosis (Fig. 2). This suggests that the absence or presence of ^18^F-FACBC uptake in lesions could be useful for response assessment and indicate residual PCNSL (Fig. 2).

Studies have shown that the evaluation of dynamic ^18^F-FET TACs can aid in differentiating low-grade from high-grade gliomas, as well as differentiating radiation necrosis from recurrence in BMs when combining slope curves with TBR (Pöpperl et al. 2007; Romagna et al. 2016; Ceccon et al. 2017; Galldiks et al. 2012). It is however uncertain whether dynamic ^18^F-FACBC can be utilized for grading gliomas or detecting radiation necrosis, this may be a result of different transport mechanisms utilized by the two tracers (Parent et al. 2020; Øen et al. 2022). With regards to dynamic analysis with ^11^C-MET, Nomura et al. found that in 31 GBMs and 8 PCNSLs they could with 100% sensitivity and 67.3% specificity distinguish PCNSLs from GBMs, by using a late phase TAC slope coefficient cut-off (Nomura et al. 2018). Okada et al. on the other hand, found that a cut-off of 1.17 in the difference between early- and late-phase SUV_max_ of dynamic ^11^C-MET uptake could differentiate perfectly between 15 GBMs and 7 DLBCLs, outperforming a static ^18^F-FDG SUV_max_ cut-off which resulted in one false-positive and one false-negative (Okada et al. 2012). In the current study, no conclusions can be drawn from dynamic TAC evaluation, though most lesions had a decreasing TBR_peak_ and increasing SUV_peak_ slope, while the two aforementioned studies describe an increasing ^11^C-MET SUV_max_ curve in PCNSLs (Okada et al. 2012; Nomura et al. 2018). Studies should be conducted to see whether there is a distinguishable difference between dynamic ^18^F-FACBC curves of PCNSLs, GBMs, and BMs, which could potentially assist physicians in diagnostic assessment.

An advantage of this study is that it shows the utility of simultaneous PET/MRI for neuroimaging. It provides neuroradiologists, nuclear medicine physicians, and oncologists with information from a single examination without the need to perform image registration, and the different image modalities are acquired under the same biological conditions. Despite being the first study to describe the use of ^18^F-FACBC in patients with PCNSLs, there are some clear limitations to this study. The evaluation of PET and MRI images was not blinded, which could have influenced PET image analysis—particularly for ^18^F-FDG images which had more tracer uptake in general. The sample size was small, resulting in a more descriptive than quantitative report. Although 5–10 patients were planned to be included in this study, there are several reasons why only three were included. Technical problems with the airplane transporting ^18^F-FACBC from Oslo to Trondheim resulted in cancellations, and study inclusion was impeded by production problems at the cyclotron lasting from October 2021 until the end of the inclusion period in December 2022. Finally, the rarity of PCNSL occurrence in addition to covid and the limitations mentioned above resulted in only three patients being included in the planned project period.

## Conclusions

^18^F-FACBC consistently provided high lesion contrast for all PET detected PCNSLs, outperforming ^18^F-FDG in the detection of PCNSLs at baseline and in response evaluation. High ^18^F-FACBC TBR values are caused by low tracer uptake in normal brain tissue—a well-established attribute which makes ^18^F-FACBC unique among AA-PET tracers. The only lesion not visually detected with ^18^F-FACBC was a small lesion which appeared atypical on ce-MRI, this could have indicated the absence of residual PCNSL as this patient was still in complete remission one year after end of treatment. Ultimately, ^18^F-FACBC may be useful as a supplement to ce-MRI in the assessment of PCNSLs, particularly for response assessment, though further studies with more subjects are required to validate these findings.

## Data Availability

The datasets generated and/or analyzed during the current study are not publicly available due to the European Union General Data Protection Regulations (GDPR), but are available from the corresponding author on reasonable request.

## Abbreviations

PCNSL: Primary central nervous system lymphomas
HD-MTX: High-dose methotrexate
ce-MRI: Contrast enhanced magnetic resonance imaging
TBRs: Tumor-to-background ratios
HGG: High-grade gliomas
BM: Brain metastases
BBB: Blood brain barrier
IPCG: International Primary CNS Lymphoma Collaborative group
PET: Positron emission tomography
^18^F-FDG: ^18^F-2-fluoro-2-deoxy-D-glucose
AA-PET: Amino acid PET
^18^F-FDOPA: 3,4-Dihydroxy-6-[^18^F]-fluoro-L-phenylalanine
^11^C-MET: [^11^C]-methyl-methionine
^18^F-FET: [^18^F]-fluoro-ethyltyrosin
RANO: Neuro-oncology working group
LAT1: L-Amino-acid-transporter 1
ASCT2: Alanine–serine–cysteine transporter 2
DLBCL: Diffuse large B-cell lymphoma
R-MPV: Rituximab, methotrexate, procarbazine and vincristine
MATRix: Methotrexate, cytarabine, rituximab, thiotepa
BCNU ASCT: Carmustine–thiotepa-conditioned autologous stem cell transplantation
IELSG score: International Extranodal Lymphoma Study Group score
MSKCC score: Memorial Sloan Kettering Cancer Center score
ECOG PS: Eastern Cooperative Oncology Group performance scale
UTE: Ultra-short echo time
VOIs: Volumes of interest
TACs: Time activity curves
TTP: Time-to-peak
